# The dual origin of the peripheral olfactory system: placode and neural crest

**DOI:** 10.1186/1756-6606-4-34

**Published:** 2011-09-23

**Authors:** Hiroyuki Katoh, Shinsuke Shibata, Kimiko Fukuda, Momoka Sato, Etsuko Satoh, Narihito Nagoshi, Takeo Minematsu, Yumi Matsuzaki, Chihiro Akazawa, Yoshiaki Toyama, Masaya Nakamura, Hideyuki Okano

**Affiliations:** 1 Department of Orthopaedic Surgery, Keio University School of Medicine, 35 Shinanomachi, Shinjuku-ku, Tokyo, 160-8582, Japan; 2 Department of Physiology, Keio University School of Medicine, 35 Shinanomachi, Shinjuku-ku, Tokyo, 160-8582, Japan; 3 Department of Biological Science, Tokyo Metropolitan University, 1-1 Minami-Osawa, Hachioji-shi, Tokyo, 192-0397, Japan; 4 Department of Gerontological Nursing/Wound Care Management, Graduate School of Medicine, The University of Tokyo, 7-3-1 Hongo, Bunkyo-ku, Tokyo, 113-0033, Japan; 5 National Institute of Musculo-Skeletal Disorders, Murayama Medical Center, 2-37-1 Gakuen, Musashimurayama-shi, Tokyo, 208-0011, Japan; 6 Department of Biophysics and Biochemistry, Graduate School of Health Sciences, Tokyo Medical and Dental University, 1-5-45 Yushima, Bunkyo-ku, Tokyo, 113-8519, Japan

**Keywords:** neural crest, olfactory placode, olfactory ensheathing cell, neural crest progenitor cell, P0-Cre, Wnt1-Cre, Sox10, chick embryo

## Abstract

**Background:**

The olfactory epithelium (OE) has a unique capacity for continuous neurogenesis, extending axons to the olfactory bulb with the assistance of olfactory ensheathing cells (OECs). The OE and OECs have been believed to develop solely from the olfactory placode, while the neural crest (NC) cells have been believed to contribute only the underlying structural elements of the olfactory system. In order to further elucidate the role of NC cells in olfactory development, we examined the olfactory system in the transgenic mice Wnt1-Cre/Floxed-EGFP and P0-Cre/Floxed-EGFP, in which migrating NC cells and its descendents permanently express GFP, and conducted transposon-mediated cell lineage tracing studies in chick embryos.

**Results:**

Examination of these transgenic mice revealed GFP-positive cells in the OE, demonstrating that NC-derived cells give rise to OE cells with morphologic and antigenic properties identical to placode-derived cells. OECs were also positive for GFP, confirming their NC origin. Cell lineage tracing studies performed in chick embryos confirmed the migration of NC cells into the OE. Furthermore, spheres cultured from the dissociated cells of the olfactory mucosa demonstrated self-renewal and trilineage differentiation capacities (neurons, glial cells, and myofibroblasts), demonstrating the presence of NC progenitors in the olfactory mucosa.

**Conclusion:**

Our data demonstrates that the NC plays a larger role in the development of the olfactory system than previously believed, and suggests that NC-derived cells may in part be responsible for the remarkable capacity of the OE for neurogenesis and regeneration.

## Background

The sensory organs of the vertebrate head derive from two embryological structures, the sensory placodes and the cranial neural crest (NC), which arise from the border between neural and non-neural ectoderms on the lateral edge of the neural plate and contribute to the formation of the peripheral sensory nervous system in an intricate relationship during cranial development. Placodes are discrete areas of thickened non-neural epithelium that form in characteristic positions in the head of vertebrate embryos and give rise to the paired sensory organs, including the olfactory system. The NC is a multipotent population of migratory cells unique to the vertebrate embryo that delaminate from the neural epithelium and migrate throughout the embryo to give rise to a wide variety of cell types [1,2].

The olfactory organ has been shown to arise from a combination of the olfactory placode and cranial NC cells, with the olfactory placode giving rise to the olfactory sensory neurons and supporting cells of the olfactory epithelium (OE) [3-5], and the NC contributing to the structural elements of the nose. The role of the olfactory placode in olfactory development was first experimentally demonstrated in the early twentieth century when resection of the olfactory placode in amphibians was shown to disrupt the development of the olfactory bulb [6]. Cell labeling and olfactory placode ablation experiments later verified that the OE develops from the olfactory placode. However it is important to note that many of these analyses were conducted in developmental stages after olfactory placode formation, and thus after anterior migration of the cranial NC cells. By the time that the placode has formed, NC cells contributing to the frontal mass have migrated anteriorly and are intimately associated with the olfactory placode. Any manipulations conducted to the olfactory placode at this stage will also affect the underlying NC cells that play an important role in olfactory development. The importance of NC cells in the development of the olfactory system was demonstrated in rSey rats with a mutation in the Pax6 gene in which impaired migration of midbrain crest cells into the frontonasal mass led to the loss of the nasal placode [7], and retinoic acid signaling from NC cells was found to be necessary for olfactory placode development [8]. The convergence of placode and NC cells in the embryo to give rise to the olfactory organ has made uncovering the developmental origins of the olfactory components very complicated.

However with the advancement of transgenic animal techniques, it has become possible to permanently label early presumptive NC cells and all subsequent progeny by using a double transgenic system. The first component is a transgene expressing Cre recombinase driven by promoters/enhancers of either Wnt1 or myelin protein zero (P0). The Wnt1 gene is expressed specifically in the neural plate, in the dorsal neural tube, and in the early migratory NC population. In NC cells, Wnt1 expression in extinguished as the cells migrate away from the neural tube, and is not expressed at any other time or place [9]. The P0 glycoprotein is a cell adhesion molecule that constitutes the myelin sheaths in the peripheral nervous system [10,11], and its mRNA has been demonstrated to be expressed in a subpopulation of NC cells after detachment from the neuroepithelium [12,13]. The second component is a reporter gene that is expressed only upon Cre-mediated recombination [14]. By observing the olfactory system in these transgenic mice, we were able to distinguish between NC-derived and olfactory placode-derived components, thus identifying the tissue origins of the olfactory organ.

In order to confirm the findings observed in the transgenic mice, we conducted cell tracing studies of NC cells in chick embryos. The *in ovo *electroporation in chick embryos has widely been used as a powerful tool to study roles of genes during embryogenesis. However, the conventional electroporation technique fails to retain the expression of transgenes for more than several days because transgenes are not integrated into the genome. To overcome this shortcoming, we utilized a transposon-mediated gene transfer method [15] and were able to observe reporter gene expression until embryonic day 13.

Examination of the olfactory system in the transgenic mice and chick embryos have revealed that NC-derived cells play a significantly larger role in olfactory development than was previously believed. Here, we demonstrate that NC-derived cells are present in both embryonic and postnatal OE with morphology and antigenic profiles that are indistinguishable from placode-derived epithelial cells, and confirm that olfactory ensheathing cells (OECs), another type of cell believed to arise from the olfactory placode [5,16,17], are also of NC lineage. We also show through the culture of olfactory mucosa-derived spheres the presence of NC progenitor cells, suggesting that NC-derived cells may in part be responsible for the remarkable capacity of the OE for neurogenesis and regeneration.

## Results

### Neural crest-derived cells in the olfactory epithelium

Transgenic mice harboring a Cre recombinase gene driven by promoters of either Wnt1 or P0 were crossed with the floxed CAG-EGFP reporter mice, generating the double transgenic mice Wnt1-Cre/Floxed-EGFP [18-20] and P0-Cre/Floxed-EGFP [21] in which cells of the NC lineage are indelibly tagged with EGFP. In both transgenic mice, GFP^+ ^cells were found in tissues known to contain NC-derived cells such as the dorsal root ganglia, sympathetic nerve ganglia, enteric nervous system, and outflow tract of the heart, and also in the stroma of various tissues including bone marrow, cornea, and kidney, confirming the effective and specific marking of the NC lineage as demonstrated in our previous studies [22-25].

**Figure 1:**
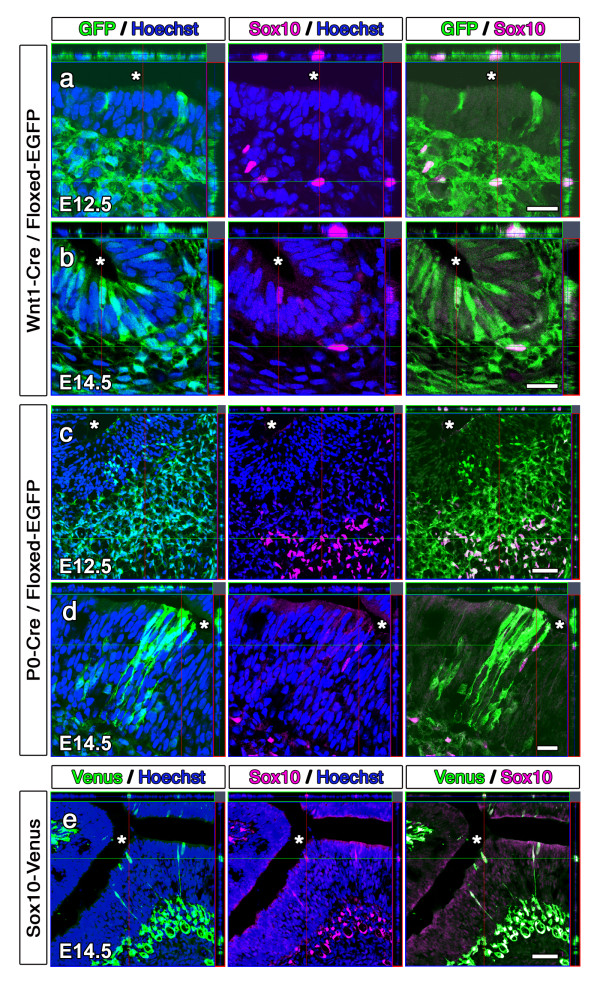
**Neural crest-derived cells in the embryonic olfactory epithelium**. **a-d**, Confocal images of direct GFP fluorescence and anti-Sox10 immunostaining in the olfactory epithelium of E12.5 (**a**) and E14.5 (**b**) Wnt1-Cre/Floxed-EGFP mice and E12.5 (**c**) and E14.5 (**d**) P0-Cre/Floxed-EGFP mice reveal the presence of neural crest-derived cells in the olfactory epithelium. **e**, Confocal images of direct Venus fluorescence and anti-Sox10 immunostaining in the olfactory epithelium of E14.5 Sox10-Venus mice confirm the presence of neural crest-derived cells in the olfactory epithelium. Asterisks indicate the nasal cavity. Scale bars: (**a**, **b**, **d**) 20 μm, (**c**, **e**) 50 μm.

The classic behavior of NC cells is to delaminate from the neural tube early in embryogenesis and directly migrate to the target tissue, where they immediately begin to differentiate into the target-appropriate cell types. We therefore examined Wnt1-Cre/Floxed-EGFP and P0-Cre/Floxed-EGFP embryos to observe the migration of NC cells into the OE. At E10.5, the olfactory placode begins to invaginate in a process that leads to the formation of the olfactory pit. In both transgenic mice at E10.5, the developing OE at this stage is mostly negative for GFP while the underlying fibronectin^+ ^mesenchyme contains numerous GFP^+ ^cells, supporting the fact that the OE mainly develops from the olfactory placode and that NC cells are present in the frontonasal mesenchyme at this stage. From E11.5, the presence of GFP^+ ^cells in the OE is observed in both Wnt1-Cre/Floxed-EGFP (Figures 1a and 1b) and P0-Cre/Floxed-EGFP mice (Figures 1c and 1d), suggesting that NC-derived cells give rise to cells of the OE. GFP^+ ^cells are found sporadically in the OE at first, and gradually increase in the embryonic epithelium until the late embryonic stage in both transgenic mice. These GFP^+ ^cells are not found uniformly throughout the OE, but rather in clusters of GFP^+ ^cells that span the epithelium.

In order to rule out ectopic expression of Wnt1 or P0 in the embryonic stage, we performed anti-Wnt1 and anti-P0 immunostaining in E10.5 and E14.5 mouse embryos. Although limited expression of both Wnt1 and P0 was observed in the mesenchyme, their expression was not observed in the developing epithelium, confirming that the GFP expression observed in the OE of Wnt1-Cre/Floxed-EGFP and P0-Cre/Floxed-EGFP mice is due to Cre-mediated recombination during ontogeny of NC cells rather than ectopic Cre expression in the epithelium. We also verified Cre-mediated recombination in GFP^+ ^cells by conducting PCR of GFP^+ ^cells collected from the olfactory mucosa through flow cytometry (Additional File 1) and confirmed the absence of GFP^+ ^cells in the OE of the reporter gene single transgenic mouse.

Since the transcription factor Sox10 is expressed in migrating NC cells and is essential for the development and differentiation of various NC-derived cell types, Sox10 immuno-reactivity is regarded as a classical NC marker. We performed immunohistochemistry for Sox10 and confirmed the expression of Sox10 in the nuclei of GFP^+ ^cells in the OE of E14.5 Wnt1-Cre/Floxed-EGFP (Figure 1b) and P0-Cre/Floxed-EGFP (Figure 1d) embryos. However, not all GFP^+ ^cells had Sox10^+ ^nuclei, and Sox10 immuno-reactivity was not observed in the OE until E13.5, suggesting that the GFP^+ ^cells of the transgenic mice and Sox10^+ ^cells may be partially overlapping but different subpopulations of NC-derived cells.

In order to further elucidate the movement of Sox10^+ ^cells into the OE and to verify the presence of NC-derived cells in the OE, we examined the embryonic OE in another transgenic mouse line, the Sox10-Venus BAC transgenic mouse (Sox10-Venus). In Sox10-Venus mice, Venus fluorescence faithfully mirrors endogenous Sox10 expression, with transient fluorescence observed in developing NC-derived tissues such as the dorsal root ganglia, melanoblasts, and Schwann cells [26]. The intensity of Venus fluorescence makes it possible to identify Sox10 expression at the single cell level. At E11.5, in which GFP^+ ^cells begin to appear in the OE of Wnt1-Cre/Floxed-EGFP and P0-Cre/Floxed-EGFP mice, Venus^+ ^cells are observed in the cranial mesenchyme but not in the OE of Sox10-Venus mice. As development progresses these Venus^+ ^cells migrate in the mesenchyme in a caudal to rostral direction and arrive in the nasal mesenchyme by E12.5. Venus^+ ^cells are found in the OE of Sox10-Venus mice by E13.5, and anti-Sox10 immunohistochemistry verifies the expression of the Sox10 protein in these Venus^+ ^OE cells (Figure 1e). The migration of Venus^+ ^cells from the lamina propria into the OE of Sox10-Venus mice verifies the results obtained by anti-Sox10 immnohistochemistry performed on Wnt1-Cre/Floxed-EGFP and P0-Cre/Floxed-EGFP mice, in which Sox10^+ ^cells are observed only in the lamina propria at E12.5, and appear in the OE by E13.5. All Sox10^+ ^cells in Wnt1-Cre/Floxed-EGFP and P0-Cre/Floxed-EGFP mice were also positive for GFP, suggesting that Sox10^+ ^NC-derived cells are a subset of the GFP^+ ^NC-derived cells identified by GFP expression in Wnt1-Cre/Floxed-EGFP and P0-Cre/Floxed-EGFP mice.

### Cell tracing studies in chick embryos

In order to confirm our observations in transgenic mice, we conducted cell tracing studies of NC cells in chick embryos to verify their migration into the OE. The chick has been used as a model system to study vertebrate development mainly due to the ease of manipulating the embryo, and the research of NC cells has been extensively conducted with chick-quail chimeras [27,28]. However, surgical ablation or transplantation of the neural fold or neural tube before onset of NC cell migration would be technically demanding and often incurs collateral damage to adjacent tissue, requiring careful consideration when interpreting the results. Furthermore, since NC cells are required for olfactory placode development [7,8], we elected to perform labelling and tracing of the NC cells and not ablation or transplantation procedures that may lead to the deformation or absence of the olfactory organ. We introduced a reporter gene into the embryo by *in ovo *electroporation, using the Tol2 transposon system that allows for continual expression of the reporter gene until late developmental stages.

**Figure 2:**
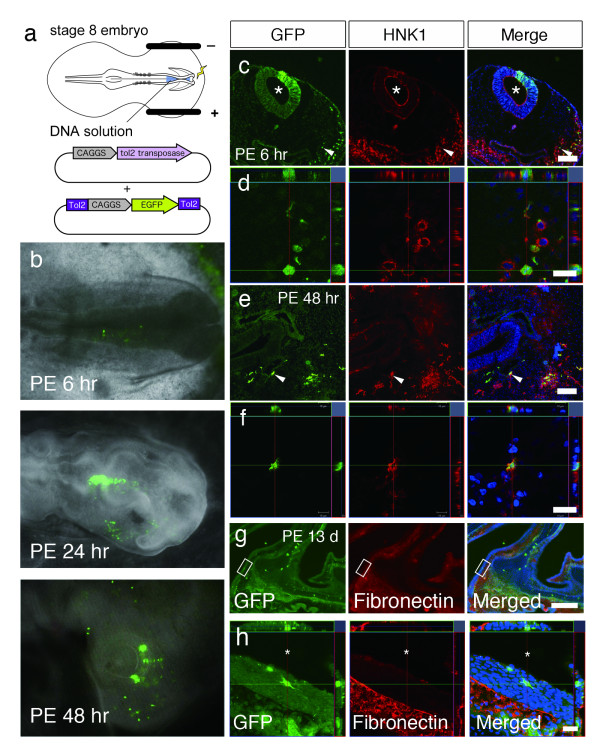
**Cell tracing study of neural crest cells in chick embryos**. **a**, Experimental schema for cell tracing of neural crest cells in the chicken embryo. Constructs were electroporated into the neural fold of stage 8 embryos at the midbrain and anterior hindbrain level before neural crest cells delaminate from the neural tube. **b**, Stereoscopic images of GFP fluorescence observed in the *in ovo *chick embryo at 6, 24, and 48 hours after introduction of GFP by electroporation into the neural fold. **c-f**, Confocal images of direct GFP fluorescence and immunostaining of HNK1 in the chick embryos at 6 hours (**c**, **d**) and 48 hours (**e**, **f**) after electroporation. The asterisks in **c **indicate the neural tube, while the arrowheads in **c **and **e **indicate the double positive cells magnified in **d **and **f**, respectively. **g,h**; Confocal images of direct GFP fluorescence in the olfactory mucosa of chick embryos at 13 days after electroporation. The boxes in **g **indicate the area magnified in **h**. Anti-fibronectin staining demarcates the mesenchyme of the olfactory mucosa and demonstrates the presence of a GFP^+ ^neural crest cell in the olfactory epithelium. The asterisks in **h **indicate the nasal cavity. Nuclei were counterstained with Hoechst. Scale bars: (**c**, **e**) 100 μm, (**d**, **f**, **h**) 20 μm, and (**g**) 200 μm.

A plasmid DNA containing the transposase cDNA under the control of a ubiquitous promoter (CAGGS) and a transposon-donor plasmid DNA containing a Tol2 construct with the CAG promoter and the gene encoding GFP [29] were introduced into the anterior neural fold of Hamburger and Hamilton stage 8 chick embryos by electroporation (Figure 2a) [15,30]. Initial GFP fluorescence was observed by post-electroporation (PE) 3 - 4 hours, and the migration of the labelled GFP^+ ^cells from the neural tube was observed by PE 6 hours. The anterior migration of the GFP^+ ^cells was observed at PE 24 hours, and GFP^+ ^cells were observed in the frontonasal and periocular areas by PE 48 hours (Figure 2b). The time frame and migration pathway of the labelled cells strongly suggest the successful labelling of the NC cells. To confirm that the migrating cells are NC cells, embryos were processed for immunohistochemistry and stained with the HNK-1 antibody, which recognizes a carbohydrate moiety that is present on the surface of migrating avian NC cells and is regarded to be a marker for avian NC cells [31]. At PE 6 hours, the area of the neural tube in which the DNA was introduced is GFP^+^, and GFP^+ ^cells are observed delaminating in streams ventrally and laterally from the dorsal tube between the neural tube and the overlying ectoderm in a course that is typical of NC cells (Figure 2c). At PE 48 hours, GFP^+ ^cells are observed in the frontonasal mesenchyme (Figure 2e). Immunohistochemistry for HNK-1 confirmed that the GFP^+ ^cells were also positive for HNK-1 in both PE 6 and 48 hour-sections (Figures 2d and 2f), demonstrating the successful labelling of the NC cells. The embryos were then allowed to sufficiently develop *in ovo*, and were sectioned for histology. At PE 13 days, a limited number of GFP^+ ^cells were found in the OE, confirming that NC cells also give rise to cells in the embryonic chick OE (Figures 2g and 2h).

### Identity of neural crest-derived cells in the olfactory epithelium

Since the observations from the transgenic mice and chick cell tracing studies revealed the presence of NC-derived cells in the OE, we performed immunohistochemistry to examine which cell types of the OE are produced by the NC. In this experiment, we performed the identification of specific epithelial cell types in postnatal animals because the identification of epithelial cells based upon their location within the OE becomes more apparent, and also to allow for complete differentiation and antigenic maturation since markers for horizonatal basal cells (HBCs) are not observed in the embryonic period.

The postnatal OE is a pseudostratified columnar epithelium overlying the lamina propria and is composed of five basic cell types that can be distinguished on morphological, biochemical, and antigenic characteristics. Deep in the epithelium are HBCs and globose basal cells (GBCs) that are regarded as the transit amplifying progenitors of the OE [32,33]. Aligned on the surface are sustentacular (SUS) cells with thin cytoplasmic projections that terminate at the basal lamina. Olfactory receptor neurons (ORNs) are situated in an intermediate zone between these basal and apical layers, and make up the bulk of the epithelium. The remaining cell type is the Bowman's gland/duct complex that extends from the glands in the lamina propria to the ducts within the epithelium, which carry the secretions to the apical epithelial surface.

**Figure 3:**
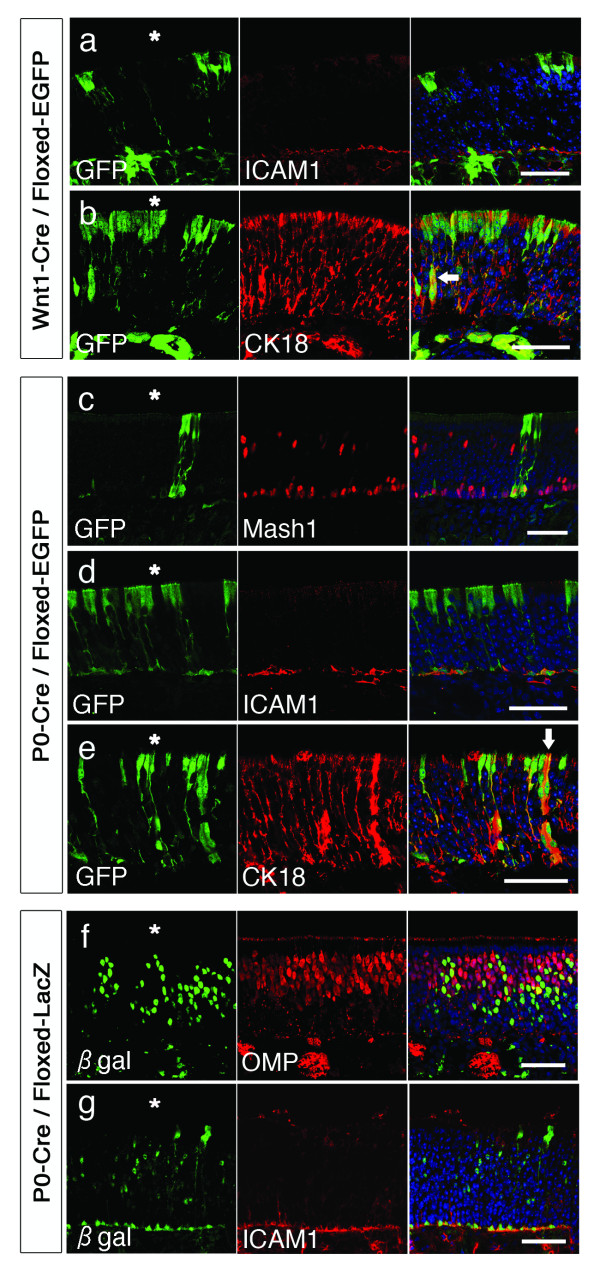
**Identification of neural crest-derived cells in the olfactory epithelium**. Confocal images of the olfactory epithelium in Wnt1-Cre/Floxed-EGFP (**a**, **b**) and P0-Cre/Floxed-EGFP (**c**-**e**) mice stained for the indicated markers reveal the presence of neural crest-derived ICAM1^+ ^HBCs, Mash1^+ ^GBCs, and CK18^+ ^SUS cells in the olfactory epithelium. All images of GFP are direct fluorescence observed in postnatal 4-week mice, except for the Mash1 staining in **c **that was observed in postnatal 2-day mice and required anti-GFP immunostaining after antigen retrieval procedures. OMP^+ ^ORNs (**f**) and ICAM1^+ ^HBCs (**g**) were observed in 4 week-old P0-Cre/Floxed-LacZ (**f**, **g**) mice. Nuclei were counterstained with Hoechst. Arrows in **b **and **e **indicate CK18^+ ^ducts of Bowman's glands and asterisks indicate the nasal cavity. Scale bars: 50 μm.

The GFP^+ ^NC-derived cells in the postnatal OE of Wnt1-Cre/Floxed-EGFP and P0-Cre/Floxed-EGFP mice had morphologies compatible with cells normally constituting the epithelium, and we conducted immunohistochemical staining for specific markers of OE cells to verify their identities. GFP^+ ^cells lining the basement membrane of the OE were mostly ICAM1^+ ^HBCs (Figures 3a and 3d). Among the GFP^+ ^cells in the basal layer, a limited number of Mash1^+ ^GBCs (Figure 3c) were identified. The GFP^+ ^cells spanning the OE were CK18^+ ^SUS cells and some Bowman's gland/ducts (Figures 3b and 3e). In both strains however, GFP^+ ^ORNs were not observed.

While the GFP^+ ^cells in the embryonic Wnt1-Cre/Floxed-EGFP and P0-Cre/Floxed-EGFP OE were distributed in clusters, this distribution was more apparent in postnatal animals. Clusters of GFP^+ ^cells including both basal and apical cells were sporadically observed, along with large areas of the OE with no apparent GFP^+ ^cells. GFP^+ ^HBCs and GBCs were observed along with SUS cells and Bowman's gland/ducts, suggesting that these transient progenitors gave rise to the SUS cells situated directly apical to their position (Figure 3).

Considering the accepted proliferation and differentiation process of the OE in which epithelial cells are replenished by transit amplifying progenitors in the basal aspect of the epithelium, we could not explain the absence of GFP^+ ^ORNs. It is possible that NC-derived cells were responsible for the replenishment/regeneration of non-neuronal cells, or that the silencing of the CAG promoter occurred in the olfactory neuronal-lineage cells. We therefore created another double transgenic mouse line by crossing P0-Cre mice with mice carrying the ROSA26 conditional reporter transgene, thereby generating mice in which NC-derived cells are labeled with β-galactosidase (P0-Cre/Floxed-LacZ). Observation of the OE in P0-Cre/Floxed-LacZ mice revealed β-galactosidase expression in a subset of ORNs (Figure 3f) and also in HBCs (Figure 3g), SUS cells, and Bowman's gland/ducts. These results demonstrated that NC-derived cells have the capability to differentiate into all cell types of the OE.

### Olfactory ensheathing cells are derived from the neural crest

The primary olfactory pathway consists of ORNs in the OE whose axons project through the cribriform plate to synapse with cells in the olfactory bulb in the central nervous system (CNS). Along the course of the nonmyelinated olfactory nerves, they are accompanied by OECs that ensheath and compartmentalize the small olfactory axons into fascicles [34]. OECs have a unique property that allows regenerating olfactory nerves to cross the peripheral/central nerve threshold and have thus been attracting interest as a potential source for transplantation to treat CNS ailments. While OECs were believed to be derived solely from the olfactory placode based on past observational studies [5,16,17], OECs display several characteristics that suggest a NC origin. OECs are peripheral glia and share multiple characteristics with Schwann cells [17]. All other peripheral glia, including Schwann cells, satellite cells, enteric glia [27], and acoustic glia [35] develop from the NC. Furthermore, OECs express p75, a NC marker expressed in all other peripheral glia.

**Figure 4:**
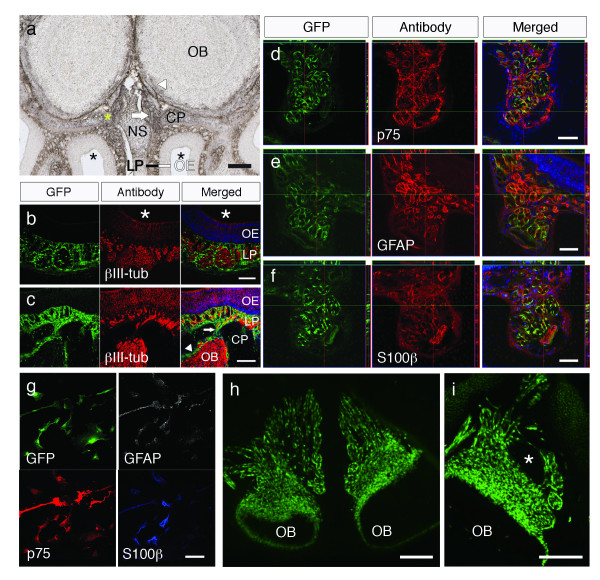
**GFP expression in OECs from Wnt1-Cre/Floxed-EGFP mice**. **a**, Anti-GFP DAB-nickel-staining of the olfactory system reveals GFP expression in areas populated by OECs. The white arrow indicates the olfactory nerve traversing the cribriform plate from the olfactory mucosa to the olfactory bulb. The white arrowhead indicates the outer layer of the olfactory bulb with GFP^+ ^cells. Note that the skeletal system and the meninges, which are derived from the neural crest, are also positive for GFP. The yellow asterisk indicates the area observed in **d**-**f**, which corresponds to the groove in the cribriform plate where the olfactory nerves pass before crossing the cribriform plate and the black asterisks indicate the nasal cavity. **b**-**c**, Confocal images of the olfactory system showing direct GFP fluorescence and immunostaining for βIII-tubulin. The arrow indicates the olfactory nerve passing through the cribriform plate and the arrowhead indicates the outer layer of the olfactory bulb with GFP^+ ^cells. **d**-**f**, Cross-section images of the olfactory nerve before it passes through the cribriform plate, displayed as Z-stack confocal images with corresponding x- and y-axes, showing GFP fluorescence and immunostaining for OEC markers p75 (**d**), GFAP (**e**), and S100β (**f**). All images are oriented with the olfactory mucosa above and the olfactory bulb below the shown area. **g**, Primary OEC cells cultured from the olfactory mucosa and verified by multiple OEC markers were positive for GFP, verifying their neural crest origin. **h-i**, Venus fluorescence in Sox10-Venus mice verifies the expression of Sox10, a neural crest marker, in OECs at E14.5 (**h**) and E15.5 (**i**) The asterisk indicates the nasal cavity. OE: olfactory epithelium, LP: lamina propria, OB: olfactory bulb, NS: nasal septum, CP: cribriform plate. Scale bars: (**a, h, i**) 200 μm, (**b**, **c**) 100 μm, (**d**-**g**) 50 μm.

To verify that OECs are derived from the NC, the olfactory mucosa of Wnt1-Cre/Floxed-EGFP mice was examined for GFP expression. P0-Cre/Floxed-EGFP mice were not used to study OECs since adult OECs have been shown to express the P0 protein *in situ *[13], but we have also confirmed that OECs are GFP^+ ^in P0-Cre/Floxed-EGFP mice (data not shown). In Wnt1-Cre/Floxed-EGFP mice, GFP^+ ^cells were found in areas known to be populated by OECs: surrounding axons in the olfactory nerve fascicles of the lamina propria (Figure 4b), accompanying olfactory nerve axons through the cribriform plate (white arrows in Figures 4a and 4c), and in the outer layer of the olfactory bulb (white arrowheads in Figures 4a and 4c). These GFP^+ ^cells were positive for OEC markers p75 (Figure 4d), GFAP (Figure 4e), and S100β (Figure 4f), indicating that these NC-derived cells are OECs. To verify GFP expression in OECs, the olfactory mucosa was dissected from Wnt1-Cre/Floxed-EGFP mice and primary OEC cells were cultured. Although we cannot exclude the possibility of OEC development from the placode or other sources, OECs identified by expression of OEC markers p75, GFAP, and S100β were GFP^+ ^(Figure 4g), demonstrating that at least a subset of OECs are derived from the NC. Furthermore, OECs in the olfactory system of embryonic Sox10-Venus mice were positive for Venus, verifying the expression of Sox10 in embryonic OECs (Figures 4h and 4i).

Recently, the NC origin of OECs was demonstrated by fate-mapping techniques in chick embryos and examination of Wnt1-Cre/ROSA-LacZ or -YFP mice [36]. Our results confirm their findings and also demonstrate that OECs cultured from Wnt1-Cre/Floxed-EGFP mice are positive for GFP. The presence of GFP^+ ^cells in the OE were attributed to the presence of Bowman's glands, but our results demonstrate that GFP expression is observed in the other cells constituting the OE.

### Neural crest progenitor cells of the olfactory mucosa

**Figure 5:**
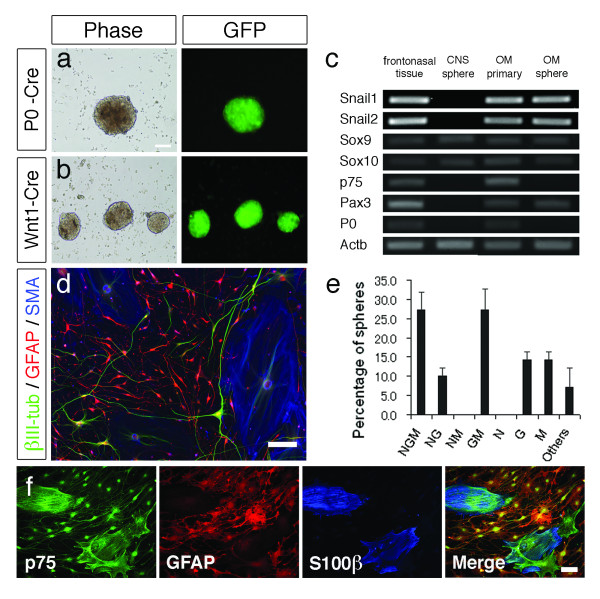
**Neural crest progenitor cells of the olfactory mucosa**. **a**, **b**, Neural crest spheres cultured from single cells of the olfactory mucosa of P0-Cre/Floxed-EGFP (**a**) and Wnt1-Cre/Floxed-EGFP (**b**) mice in medium containing 1% methlylcellulose. **c**, RT-PCR analysis for the indicated neural crest markers from the following: E14.5 frontonasal tissue containing neural crest cells (frontonasal tissue), neurospheres prepared from E14.5 striatum of P0-Cre/Floxed-EGFP mice (CNS sphere), flow-cytometry-sorted GFP^+ ^cells of the olfactory mucosa (OM primary), and cultured spheres from the olfactory mucosa (OM sphere) of postnatal 4 week P0-Cre/Floxed-EGFP mice. **d**, Neurons, glial cells, and myofibroblasts differentiated from clonal olfactory mucosa spheres, confirming the multipotency of the cells constituting these spheres. **e**, Cells from clonally cultured olfactory mucosa spheres were differentiated and stained for cell type markers: βIII-tubulin^+ ^neurons, GFAP^+ ^glial cells, and SMA^+ ^myofibroblasts. The frequency of spheres that differentiated solely into neurons (N), glia cells (G), myofibroblasts (M) or into a combination of the three is presented as a percentage of the total number of spheres examined. Each bar represents the mean ± SD of three independent experiments, counting at least 20 spheres per experiment. **f**, Immunocytochemistry of cells differentiated from an olfactory mucosa sphere for OEC markers. Scale bar: (**a**) 50 μm, (**d**) 100 μm., (**f**) 100 μm.

The NC has recently been receiving great interest, with reports of sphere-forming NC stem/progenitor cells being isolated and cultured from numerous tissues containing NC-derived cells such as the skin [37], whisker follicles [38], heart [23], adipose tissue [39], cornea [22], and bone marrow [24]. Since our results demonstrated the presence of NC-derived cells in the OE, we examined spheres cultured from the olfactory mucosa for NC progenitor cells, suspecting that the multipotency of neurospheres previously described to be cultured from the OE [40,41] may be due to the presence of NC progenitor cells. By culturing dissociated cells of the olfactory mucosa in medium containing epidermal growth factor (EGF) and fibroblast growth factor-2 (FGF-2), large populations of floating spheres were obtained by 3 to 4 weeks. Spheres were cultured from both the OE and lamina propria when an attempt was made to divide and culture these segments separately. However, since a complete separation of the OE from the lamina propria is technically impossible, we decided to dissociate and culture the whole olfactory mucosa. To determine the characteristics of these sphere-forming cells, we performed clonal density sphere cultures in medium containing 1% methylcellulose. This method was demonstrated to be effective in preventing sphere fusion [22,24], with over 90% of the generated spheres being clonal (Additional File 2). Spheres cultured from the olfactory mucosa of both Wnt1-Cre/Floxed-EGFP and P0-Cre/Floxed-EGFP mice were GFP^+^, revealing that the sphere-forming cells are derived from the NC (Figures 5a and 5b), and immunohistochemistry of these spheres revealed Nestin expression. Passage of these spheres generated secondary and tertiary spheres, demonstrating self-renewal of the cells constituting the spheres. To examine the RNA profile of these spheres for expression of NC lineage markers, RT-PCR analysis of the following samples cultured/obtained from P0-Cre/Floxed-EGFP mice was performed: spheres cultured from the olfactory mucosa (OM sphere), GFP^+ ^primary olfactory mucosa cells sorted by flow-cytometry (OM primary), primary neurospheres cultured from the striatum of neonates (CNS sphere), and tissue collected from the frontonasal area (frontonasal tissue) which was included as a positive control for NC cells (Figure 5c). All of the examined NC markers were detected in olfactory mucosa spheres, OM primary cells, and frontonasal tissue cells. Olfactory mucosa spheres did not express P0 or p75, but P0-expression was detected in primary olfactory mucosa cells due to the presence of OECs.

To examine the multipotency of the cells contained in olfactory mucosa spheres, we performed differentiation assays of clonal spheres and compared the results with that of clonal spheres cultured from the dorsal root ganglion, a tissue in which NC stem/progenitor cells are known to exist [24,42]. Clonal spheres were individually collected, plated in differentiation medium for 10 days, and stained to identify the three types of cells reported to be generated by NC stem/progenitor cells: βIII-tubulin^+ ^neurons (N), GFAP^+ ^glial cells (G), and SMA^+ ^myofibroblasts (M) [43]. Olfactory mucosa spheres demonstrated a trilineage (NGM) differentiation potential (27.1 ± 4.7%) (Figures 5d and 5e), suggesting the presence of NC progenitor cells. This proportion was lower than that of dorsal root ganglion spheres (74.6 ± 1.0%) which generally demonstrate a neuron-dominant differentiation pattern [24]. On the other hand, olfactory mucosa spheres demonstrated a glia-dominant differentiation pattern (GM 27.1 ± 5.5%; NG 10.0 ± 2.2%; G 14.3 ± 2.1%; M 14.3 ± 2.1%; no NM or N). Cell-intrinsic differences have been shown to determine the differentiation characteristics of NC stem/progenitor cells [44], and our results indicate that the olfactory mucosa contains NC stem progenitors that are predisposed to a glial lineage. Furthermore, immunocytochemistry of differentiated cells revealed the presence of cells that were positive for OEC markers (Figure 5f), suggesting that the NC progenitor cells of the olfactory mucosa spheres are able to produce OECs. Most cells were found to be p75^+^, possibly reflecting their NC origin, since p75 has consistently been observed to mark NC stem cells in the gut [45-47] and other tissues. Cells double positive for p75 and S100β generally had a flat morphology while p75 and GFAP double-positive cells had spindly bipolar, tripolar, or stellate morphologies.

## Discussion

The olfactory placode has been described as one of the most versatile placodes, being unique in its capacity to give rise to glial cells and stem cells capable of generating various differentiated cell types of the OE throughout life. However our findings suggest that the unique properties that have been attributed solely to the olfactory placode may in part be properties endowed by NC-derived cells. In this study, we demonstrate the presence of postmigratory NC progenitor cells within the olfactory mucosa and show that OECs are derived from the NC. Furthermore, even though the majority of cells in the OE are derived from the olfactory placode, we demonstrate the capacity of NC-derived cells to give rise to all cell types of the OE.

The OE, with its capacity for continual neurogenesis in adults, has long been a hotspot for the study of neuroscience. However, identifying different progenitors in the embryonic or adult OE has been historically challenging due to the difficulty in distinguishing distinct embryonic and adult olfactory epithelial progenitors, expanding them *in vitro*, and demonstrating their developmental potential *in vivo*. With the development of sophisticated transgenic mouse technologies, it has become possible to demonstrate the relationship between progenitors and its descendants and to temporally map the progeny derived from a progenitor in fine developmental time windows. By utilizing transgenic mice that express Cre recombinase behind one of two promoters (derived from the Wnt1 or P0 gene) known to be active in migrating NC cells, we demonstrated the presence of NC-derived cells in the embryonic and postnatal OE.

Clusters of GFP^+ ^cells spanning the epithelium were observed from E10.5, and generally gradually increased in number during the embryonic period. In the mouse OE, neurogenesis during embryonic establishment (E10 - E18.5), postnatal expansion (P0 - P30), and adult maintenance (P30-) proceed with distinct, spatiotemporal patterns and different cellular and extracellular environments. During embryonic OE development, there is a dramatic expansion of epithelial cells that is brought about by proliferating cells that are equally distributed between the apical and basal OE during early embryonic stages, but become largely located at the basal area by late embryonic stages [48]. The observed increase in GFP^+ ^cells during the embryonic period suggests that NC-derived cells may support cell expansion during the early embryonic period.

We found that a subset of cells in the embryonic olfactory mucosa expressed Sox10, which has been shown to be required for the survival and maintenance of multipotent NC cells. Sox10 is also required for specification of NC cells to the glial lineage, since peripheral glia is absent in Sox10-deficient mice [49,50]. Further studies are required to elucidate the characteristics of the Sox10-expressing cells in the olfactory system, but these cells may be multipotent NC progenitor cells or mature olfactory cells that have differentiated into OECs or SUS cells, because Sox10 has been shown to be expressed in Schwann cells after specification to the glial lineage [51].

The presence of scattered clusters of GFP^+ ^cells in the postnatal mice OE with morphologic and antigenic properties identical to olfactory placode-derived epithelial cells demonstrate that NC-derived cells retain the potential to give rise to epithelial cells in adults. Unlike the NC-derived cells in the embryonic OE that seem to participate in the expansion of OE cells, NC-derived cells in the postnatal OE were often observed as clusters of GFP^+ ^cells. The clusters of GFP^+ ^cells were often comprised of HBCs, SUS cells, and Bowman's gland/duct cells, and the presence of GFP^+ ^GBCs along with β-galactosidase^+ ^ORNs in P0-Cre/Floxed-LacZ mice were also observed. HBCs are relatively quiescent, and do not take part in the normal maintenance of the OE or the replenishment of the OE after olfactory bulbectomy, in which GBCs lead the repopulation effort. However in extensive epithelial lesions following methlyl bromide exposure, HBCs demonstrate a multilineage potential that regenerates all cell types of the epithelium [33]. Since GFP^+ ^HBCs were frequently observed in the sporadic GFP^+ ^cell patches of the postnatal Wnt1-Cre/Floxed-EGFP and P0-Cre/Floxed-EGFP mice OE, NC-derived cells may give rise to HBCs that later generate other cells of the epithelium. This hypothesis may be examined through methlyl bromide lesion experiments with Wnt1-Cre/Floxed-EGFP and P0-Cre/Floxed-EGFP mice. The fact that HBCs in culture have demonstrated the capacity to generate OECs along with neurons and other non-neural cells verifies another characteristic of HBCs that may be related to the NC [52].

The multipotent developmental capacity of NC cells to differentiate into both neuronal and mesenchymal derivatives [22,53-55] has resulted in the NC being considered a fourth germ layer. The multipotency and self-renewal of NC cells from various embryonic sources *in vitro *demonstrated the presence of NC stem/progenitor cells. With the discovery of multipotent NC stem/progenitor cells not only in late gestation embryonic tissues but also in adults, the possibility of therapeutic applications has initiated intense studies to identify and characterize NC stem/progenitor cells in multiple NC-derived adult tissues. Here, we demonstrate the capacity of NC-derived cells of the olfactory mucosa to generate spheres *in vitro*, a characteristic of proliferative NC-derived cells. Furthermore, when placed on serum-containing differentiation medium, these spheres exhibited a trilineage differentiation potential by giving rise to neurons, glial cells, and myofibroblasts, thus indicating the presence of NC progenitor cells in the olfactory mucosa. The frequency of olfactory mucosa spheres with a trilineage potential was 27.1%, which is considerably lower than that of NC progenitor cells derived from the dorsal root ganglion (74.6%). A series of our recent reports on NC stem/progenitor cells has shown that their self-renewal capacity is reflective of their differentiation capacity [24], providing a possible explanation for the limited expansion capacity of olfactory mucosa-derived NC progenitor cells compared to dorsal root ganglion-derived NC progenitor cells. The differentiation characteristic of olfactory mucosa-derived NC progenitor cells demonstrated a glia-dominant differentiation pattern, confirming the *in vivo *data from the transgenic mice demonstrating the higher tendency of NC-derived cells to give rise to OECs and SUS cells. Although we have not examined the relationship between the olfactory mucosa-derived NC progenitor cells that we isolated and the progenitors of the OE, our study suggests the possibility that cells derived from the NC may be involved in the remarkable regenerative capacity of the OE.

The isolation of NC-derived progenitor cells from the olfactory mucosa, with an established method for amplification as spheres and the ability to differentiate into OECs, opens the door to possible clinical applications. OECs, with the unique ability to guide axons into the CNS, have attracted the attention of researchers studying therapy strategies for neurotrauma, and cultured OECs have been shown to be beneficial for the treatment of peripheral and CNS ailments [56,57]. NC-derived stem cells identified in numerous tissues have rapidly asserted its presence as a potential source for transplantation. Unlike embryonic stem cells that are hindered by ethical issues, NC-derived stem cells allow for autologous transplantation, and several NC-derived stem cells have been demonstrated to have a beneficial effect after transplantation into peripheral and CNS lesions. One of the well-documented and promising candidates is the NC-derived stem cell found in the hair follicle. Nestin^+ ^stem cells isolated from the hair follicle can be cultured as spheres, and have demonstrated the ability to differentiate into neurons, glia, keratinocytes, smooth muscle cells, and melanocytes *in vitro *[58-61]. Subsequent studies showed that transplanted hair follicle stem cells could enhance the regrowth and functional rejoining of the severed sciatic and tibial nerves [62], as well as the severed spinal cord [63,64], in immunocompetent mice. Although the capacity to generate OECs makes olfactory mucosa-derived NC progenitor cells an appealing transplantation candidate, the studies conducted with hair follicle stem cells clarifies the futures studies required to investigate the characteristics and therapeutic potential of this progenitor cell.

The cephalic sensory system develops through an intricate collaboration between sensory placodes and NC cells, and our results demonstrate that the role NC cells play in the development of the olfactory system is greater than previously reported. Our findings indicating a dual origin for cells of the OE inspire a resurgence of developmental research, and provide further evidence of the versatility and morphogenic capacity of NC cells.

## Methods

### Mouse lines

**The **P0-Cre mouse was obtained from Dr. K. Yamamura (Kumamoto University, Japan). Wnt1-Cre mouse and LacZ mice were purchased from The Jackson Laboratory. The CAG-CAT-EGFP mouse was obtained from Dr. J. Miyazaki (Osaka University, Japan). Wnt1-Cre and P0-Cre mice were crossed with CAG-CAT-EGFP transgenic mice to produce Wnt1-Cre/Floxed-EGFP and P0-Cre/Floxed-EGFP mice, respectively. P0-Cre mice were crossed with LacZ mice to produce P0-Cre/Floxed-LacZ mice. All experimental procedures and protocols for animals conformed to the National Institutes of Health Guide for the Care and Use of Laboratory Animals and were approved by the Animal Care and Use Committees of Keio University.

### Immunohistochemistry

Embryonic mice were anesthetized in ice, decapitated, and fixed overnight in 4% paraformaldehyde (PFA) in 0.1 M phosphate buffered saline (PBS). After sequential treatment in a graded series of 10% and 30% sucrose in PBS at 4°C, the head was embedded in optimal cutting temperature (OCT) compound and sectioned on a cryostat at 15 μm. All mice over 2 weeks of age were deeply anesthetized, transcardially perfused with 4% PFA in 0.1 M PBS and decapitated. After postfixation in 4% PFA overnight at 4°C, the skull was decalcified for 7 days in 0.5 M EDTA (Decalcifying Solution B, Wako), and sequentially soaked in a graded series of 10% and 30% sucrose in PBS at 4°C. The head was embedded in OCT compound and sectioned on a cryostat at 20 μm.

Immunostaining was performed with the following primary antibodies: rabbit anti-fibronectin (Chemicon), mouse anti-Mash1 (R&D Systems), mouse anti-cytokeratin 5/14 (Chemicon), mouse anti-cytokeratin 18 (Chemicon), goat anti-olfactory marker protein (OMP, Wako), mouse anti-βIII-tubulin (TuJ-1, Babco), rabbit anti-glial fibrillary acidic protein (GFAP, Dako), mouse anti-α smooth muscle actin (SMA, Sigma-Aldrich), rabbit anti-p75 low affinity nerve growth factor-receptor (p75, Chemicon), mouse anti-S100β (Sigma-Aldrich), and rabbit anti-βgal (Sigma). The competence of all immunohistochemisry procedures was confirmed with a negative control in which primary antibodies were omitted. Staining of Mash1 required an antigen retrieval procedure with 10 min 105°C autoclave in Target Retrieval Solution (TRS, Dako) followed by indirect immunoperoxidase amplication procedures using ABC-Elite (Vector Laboratories) and TSA (Perkin Elmer), in which case GFP was stained with anti-GFP polyclonal antibody (Chemicon). Staining of βgal was also amplified with TSA (Perkin Elmer). Nuclei were counterstained with Hoechst 33342 (Molecular Probes). Images were obtained by fluorescence microscopy (Axioskop 2 Plus, Carl Zeiss) or confocal microscopy (LSM700, Carl Zeiss) and assembled using Adobe Photoshop.

### Cell tracing of neural crest cells in chick embryo

Fertilized eggs were incubated at 38°C in a humid incubator to stage 8 according to Hamburger and Hamilton [65]. Embryos were exposed by making an opening at the sharp edge of the egg shell. DNA solution including 2 mg/ml pCAGGS-TP, 4 mg/ml pT2AL200R175CAGGFP [29], and 1% fast green in TE buffer was injected to the anterior neural fold, which will give rise to the midbrain and anterior hindbrain. A pair of platinum electrodes CUY611P3-1 (Nepagene, Ichikawa, Japan) was placed on the vitelline membrane beside the embryos. Five square pulses (9 V, 25 msec each) after a high-voltage pulse (50 V, 0.05 msec) were charged by an electroporator CUY21EX (Bex, Tokyo, Japan). After electroporation, the opening was sealed with Scotch tape (Scotch 313), and the embryos were reincubated at 38°C until the desired stages. The embryos were dissected, fixed in 4% PFA overnight at 4°C, and sequentially soaked in a graded series of 10% and 30% sucrose in PBS at 4°C. The embryo was embedded in OCT compound and sectioned on a cryostat at 20 μm.

### Olfactory mucosa cell preparation

Animals were deeply anesthetized, killed by cervical dislocation and decapitated. The olfactory mucosa was carefully dissected into ice-cold media hormone mix (MHM) [66], washed twice in Hanks' Balanced Salt Solution (HBSS, calcium- and magnesium-free; Invitrogen) and incubated for 20 min at 37°C in Dispase II (Boehringer Mannheim). The tissue was then digested in 0.5% collagenase type IA (Sigma-Aldrich) in MHM for 20 min at 37°C and mechanically dissociated. Cells were passed through an 80 μm cell-strainer-mesh and resuspended in MHM.

### Primary OEC preparation

The olfactory mucosa was treated as above with Dispase II and the superficial portion of the epithelium was shed off. The remaining tissue was collected in HBSS and centrifuged. The cell pellet was digested in 0.5% collagenase type IA in DMEM/F12 for 20 min at 37°C and dissociated. The cells were collected in DMEM/F12 +10% fetal bovine serum (FBS), spun down, and resuspended in culture medium. The culture medium consisted of DMEM/F12 supplemented with 10% FBS. Cells were fixed with 4% PFA for 10 min for immunostaining.

### Sphere forming cultures

Olfactory mucosa cells were resuspended in MHM [66] supplemented with recombinant human EGF (100 ng/ml, Pepro Tech), recombinant human FGF-2 (100 ng/ml, Pepro Tech), B27 supplement (Invitrogen), and Antibiotic-Antimycotic (Invitrogen). For clonal sphere formation, cells were plated at a density of 8.0 × 10^4 ^cells/well (6-well plates) in culture medium containing 1% methylcellulose. Spheres from CNS were prepared as described previously [67].

### Clonal analysis

Each sphere was individually plated in a chamber of poly-D-lysin/laminin (Sigma-Aldrich/Invitrogen)-coated 8-well chamber slides (Iwaki) and cultured for 14 days in MHM supplemented with 10% FBS, without any growth factors. Differentiated cells were fixed with 4% PFA in PBS, boiled for 5 min to inactivate GFP-fluorescence, and pretreated with PBS containing 0.3% Triton-X100 for 5 min at room temperature before immunostaining. The samples were mounted and observed with a universal fluorescence microscope (Axioskop 2 Plus, Carl Zeiss).

### RT-PCR

RNA was prepared using Trizol (Invitrogen) for primary GFP^+ ^cells sorted from the olfactory mucosa, and RNeasy mini kit (Qiagen) for the other samples. RNA was treated with RNase-free DNase and cDNA was generated using oligo (dT) primers and SuperScript II RT reverse transcriptase (Invitrogen) as directed by the manufacturer. The following primers were obtained from Takara: Snail1, MA030195; Snail2, MA030179; p75, MA004379; Pax3, MA026554; p0, MA026318; and Actb, MA023938. For detection of Sox9 and Sox10, primers were designed as follows: Sox9-F, 5'-CAAGTGTGTGTGCCGTGGATAG-3'; Sox9-R, 5'-CCAGCCACAGCAGTGAGTAAGAA-3'; Sox10-F, 5'-ACGCACTGAGGACAGCTTTGA-3'; and Sox10-R, 5'-ATGAGGTTATTGACAGCTTTGA-3'.

### Flow-cytometric isolation of GFP^+ ^cells

P0-Cre/Floxed-EGFP mice (postnatal 4 weeks) were killed and olfactory mucosa cells were collected as described above. Cells were stained with PI and subjected to flow-cytometry using Vantage (BD Biosciences) to sort out GFP^+ ^cells and eliminate PI^+ ^dead cells. Collected cells were immediately subjected to RNA preparation.

## Abbreviations

OE: olfactory epithelium; OEC: olfactory ensheathing cell; NC: neural crest; P0: protein zero; (E)GFP: (enhanced) green fluorescent protein; p75: p75 low affinity nerve growth factor receptor; PE: post-electroporation; HBC: horizontal basal cell; GBC: globose basal cell; SUS: sustentacular (cell); ORN: olfactory receptor neuron; CNS: central nervous system: GFAP: glial fibrillary acidic protein; EGF: epidermal growth factor; FGF: fibroblast growth factor; OM: olfactory mucosa; SMA: smooth muscle actin; PFA: paraformaldehyde; OCT: optimal cutting temperature; PBS: phosphate buffered saline; MHM: media hormone mix; DMEM: Dulbecco's Modified Eagle's Medium; FBS: fetal bovine serum.

## Competing interests

The authors declare that they have no competing interests.

## Authors' contributions

HK, SS, NN, KF, ES, MS, and TM performed the experiments. KF, ES, MS, and TM performed cell tracing studies in chick embryos. CA provided the Sox10-Venus mice. YM supported all flow cytometry analyses. YK, AT, and YT participated in the preparation of this study. HK, SS, ES, MN, and HO wrote the manuscript. HO provided financial support for the experiments. All authors have read and approved the manuscript.

## Supplementary Material

Additional file 1 **PCR confirms Cre recombination in GFP^+ ^cells**. **a**, Schematic diagrams of the CAG-CAT-EGFP transgene cassettes in the transgenic mice. The Cre recombinase excises the loxP-flanked CAT reporter gene resulting in GFP expression. The arrows under the gene constructs indicate the position and direction of the primers used for PCR. pA is a polyadenylation signal. The sizes of the PCR products are indicated under each gene construct. **b **Genomic PCR of GFP-positive or negative cells sorted from the olfactory mucosa of P0-Cre/Floxed-EGFP mice with the primer sets shown in **a **confirms Cre-mediated recombination in GFP^+ ^cells. The sequence of the primers are: CAGp F, 5'-CTGCTAACCATGTTCATGCC-3'; EGFP R1, 5'-TGGTGCAGATGAACTTCAGG-3'; EGFP F, 5'-AGCACGACTTCTTCAAGTCC-3'; EGFP R2, 5'-TGAAGTTCACCTTGATGCCG-3'. Click here for file

Additional file 2 **Clonal sphere culture in medium containing 1% methylcellulose**. To determine cell density required for clonal culture, cells from the olfactory mucosa of GFP and RFP mice were equally mixed and cultured at a density of 8.0 × 10^4 ^cells or 1.6 × 10^5 ^cells per well in 6-well plates. The culture medium contained 1% methylcellulose to inhibit sphere migration and fusion. **a**, Formed spheres. Most spheres were of a single color but a small percentage of spheres were mixed (2.3 ± 4.0% at 8.0 × 10^4 ^cells/well, 14.7 ± 8.5% at 1.6 × 10^5 ^cells/well). **b**, Percentage of single and mixed-color spheres. Values represent mean ± SD (n = 3). Click here for file
