# Supplementary figures and images for: The dual origin of the peripheral olfactory system: placode and neural crest
Source: Mol Brain. 2011 Sep 23;4:34. doi: 10.1186/1756-6606-4-34 (PMC3215936; doi:10.1186/1756-6606-4-34)

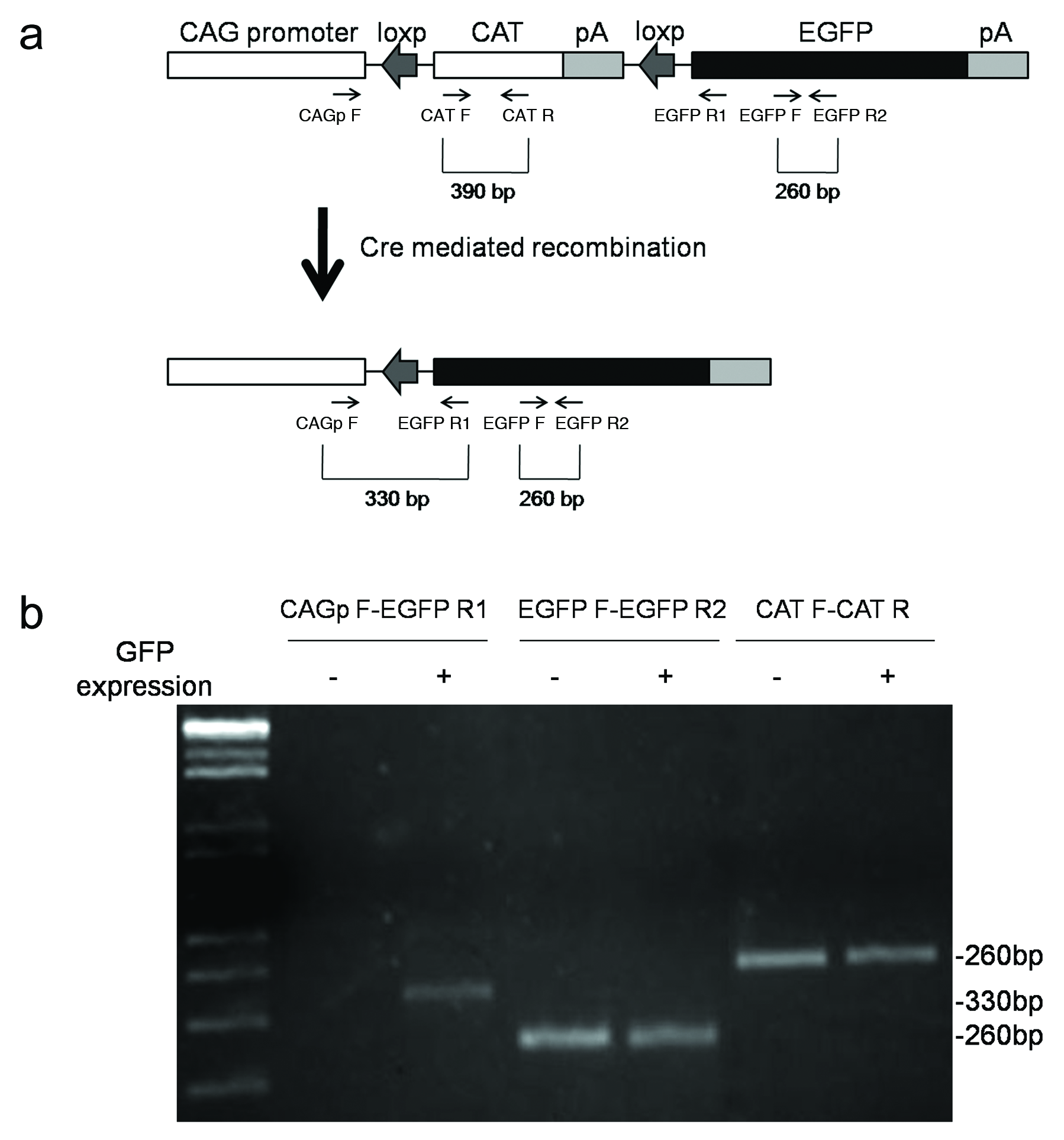

Supplement: Additional file 1 — PCR confirms Cre recombination in GFP+ cells. a, Schematic diagrams of the CAG-CAT-EGFP transgene cassettes in the transgenic mice. The Cre recombinase excises the loxP-flanked CAT reporter gene resulting in GFP expression. The arrows under the gene constructs indicate the position and direction of the primers used for PCR. pA is a polyadenylation signal. The sizes of the PCR products are indicated under each gene construct. b Genomic PCR of GFP-positive or negative cells sorted from the olfactory mucosa of P0-Cre/Floxed-EGFP mice with the primer sets shown in a confirms Cre-mediated recombination in GFP+ cells. The sequence of the primers are: CAGp F, 5'-CTGCTAACCATGTTCATGCC-3'; EGFP R1, 5'-TGGTGCAGATGAACTTCAGG-3'; EGFP F, 5'-AGCACGACTTCTTCAAGTCC-3'; EGFP R2, 5'-TGAAGTTCACCTTGATGCCG-3'. [file 1756-6606-4-34-S1.TIFF]

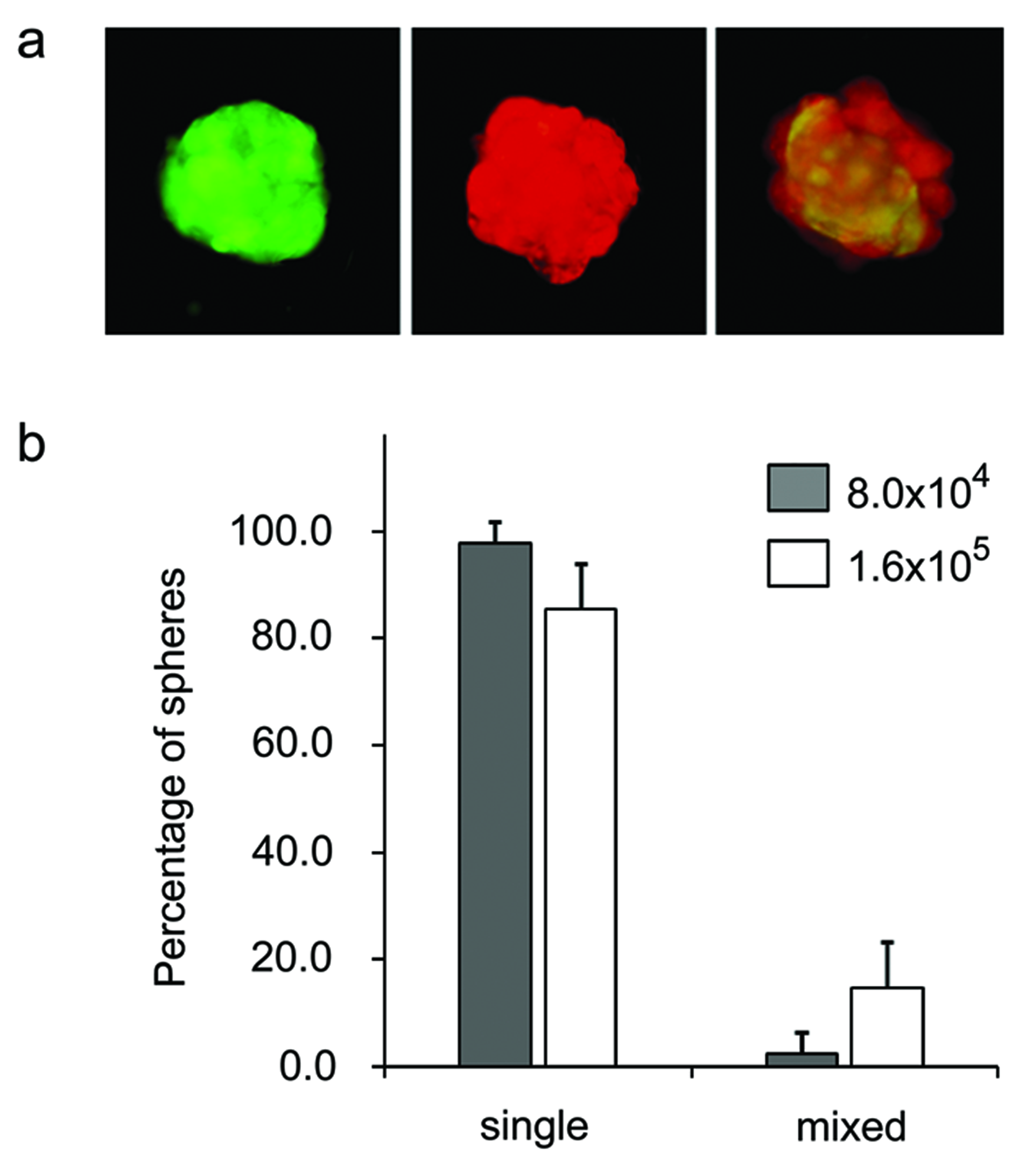

Supplement: Additional file 2 — Clonal sphere culture in medium containing 1% methylcellulose. To determine cell density required for clonal culture, cells from the olfactory mucosa of GFP and RFP mice were equally mixed and cultured at a density of 8.0 × 10^4^ cells or 1.6 × 10^5^ cells per well in 6-well plates. The culture medium contained 1% methylcellulose to inhibit sphere migration and fusion. a, Formed spheres. Most spheres were of a single color but a small percentage of spheres were mixed (2.3 ± 4.0% at 8.0 × 10^4^ cells/well, 14.7 ± 8.5% at 1.6 × 10^5^ cells/well). b, Percentage of single and mixed-color spheres. Values represent mean ± SD (n = 3). [file 1756-6606-4-34-S2.TIFF]
